# Interventions Facilitating Family Communication of Genetic Testing Results and Cascade Screening in Hereditary Breast/Ovarian Cancer or Lynch Syndrome: A Systematic Review and Meta-Analysis

**DOI:** 10.3390/cancers13040925

**Published:** 2021-02-23

**Authors:** Vasiliki Baroutsou, Meghan L. Underhill-Blazey, Christian Appenzeller-Herzog, Maria C. Katapodi

**Affiliations:** 1 Department of Clinical Research, Faculty of Medicine, University of Basel, 4055 Basel, Switzerland; vasiliki.baroutsou@unibas.ch; 2 School of Nursing, Wilmot Cancer Institute Hereditary Cancer Program, University of Rochester, Rochester, NY 14642, USA; Meghan_Blazey@URMC.Rochester.edu; 3 University Medical Library, University of Basel, 4051 Basel, Switzerland; christian.appenzeller-herzog@unibas.ch

**Keywords:** Tier-1 genetic conditions, intervention efficacy, randomized controlled trials, psychoeducational interventions

## Abstract

**Simple Summary:**

In general, 5–20% of all cancers are due to pathogenic variants in cancer genes that are passed down in the family. It is recommended that blood relatives of individuals with such a pathogenic variant have genetic testing, to identify if they also carry the same variant. This information will help their healthcare providers to make individualized cancer screening and prevention plans. However, only around 30% of at-risk relatives have genetic testing, presumably due to a lack of communication about inherited cancer genes among family members. In this paper, we identified interventions that were designed to improve family communication about hereditary cancer and/or genetic testing among at-risk relatives for two common hereditary cancer syndromes. We analyzed the components of these interventions and synthesized outcomes with statistical methods. Although we identified 14 eligible studies, there are still many unanswered questions about clinical and research implications with diverse samples to be addressed in future studies.

**Abstract:**

Evidence-based guidelines recommend cascade genetic testing of blood relatives of known Hereditary Breast and Ovarian Cancer (HBOC) or Lynch Syndrome (LS) cases, to inform individualized cancer screening and prevention plans. The study identified interventions designed to facilitate family communication of genetic testing results and/or cancer predisposition cascade genetic testing for HBOC and LS. We conducted a systematic review and meta-analysis of randomized trials that assessed intervention efficacy for these two outcomes. Additional outcomes were also recorded and synthesized when possible. Fourteen articles met the inclusion criteria and were included in the narrative synthesis and 13 in the meta-analysis. Lack of participant blinding was the most common risk of bias. Interventions targeted HBOC (*n =* 5); both HBOC and LS (*n =* 4); LS (*n =* 3); or ovarian cancer (*n =* 2). All protocols (*n =* 14) included a psychoeducational and/or counseling component. Additional components were decision aids (*n =* 4), building communication skills (*n =* 4), or motivational interviewing (*n =* 1). The overall effect size for family communication was small (*g =* 0.085) and not significant (*p =* 0.344), while for cascade testing, it was small (*g =* 0.169) but significant (*p =* 0.014). Interventions show promise for improving cancer predisposition cascade genetic testing for HBOC and LS. Future studies should employ family-based approaches and include racially diverse samples.

## 1. Introduction

Breast, colorectal, ovarian, and endometrial cancers constitute around 30% of newly diagnosed cancer cases [1,2]. In general, it is considered that approximately 5–10% of all breast and approximately 20% of ovarian cancer cases are due to an inherited pathogenic variant associated with Hereditary Breast and Ovarian Cancer (HBOC) syndrome, with some estimates being higher for selected patients and families [3,4,5,6,7]. Lynch Syndrome (LS) accounts for 2–5% of colorectal and endometrial cancer cases and is associated with increased risk for several other malignancies, including pancreatic, gastric, ovarian, and small bowel cancer [8,9,10]. Individuals with HBOC or LS are more likely to develop cancer, usually before the age of 50, at which routine cancer screening applies [11].

Germline pathogenic variants associated with HBOC and LS are inherited in an autosomal dominant manner. First- and second-degree relatives and first cousins have 12.5–50% probability of inheriting the respective cancer predisposition. The availability of cancer genetic services (counselling and testing) for “actionable” hereditary cancer syndromes, such as HBOC and LS, is a significant milestone for effective cancer prevention and control [12,13]. When a pathogenic variant is identified, relatives can be tested with 100% accuracy. Intensive surveillance starting at a younger age, prophylactic surgery, and chemoprevention can lower the risk of primary and secondary cancers, reducing morbidity and mortality for those who carry the pathogenic variant and medical and insurance costs for those who test negative [14]. The Centers for Disease Control and Prevention (CDC), Office of Public Health Genomics, USA, issued evidence-based recommendations for genetic testing in affected individuals and unaffected relatives when there is a known family history of HBOC, personal history of *BRCA*-related cancers, and LS-related colorectal cancer [15,16]. Cascade genetic screening means identifying and testing blood relatives of mutation carriers to determine if they also carry the pathogenic variant and propose risk management options [13].

Despite calls to action for HBOC and LS cascade genetic testing, there are systemic barriers to its implementation. Privacy laws worldwide prohibit healthcare providers from revealing genetic information to anyone except the tested individual. The responsibility to share genetic test results lies almost exclusively with the mutation carrier, who has the right not to disclose this information [17,18]. This communication strategy has significant limitations in both ensuring contact with the appropriate people and the transmission of accurate information [19,20]. Potential benefits of genetic testing are not being effectively communicated through family networks, leading to more than 50% of at-risk individuals not using genetic services [21]. Nevertheless, a family-based approach in communicating hereditary cancer risk is advantageous because it is not limited only to those in contact with the healthcare system but may reach relatives through the social functions already existing within the family network [22]. Interventions that support mutation carriers during the disclosure of genetic test results can reduce their psychological distress and provide relatives with accurate and credible information about cascade genetic testing. Technology-enabled education is not inferior to face-to-face genetic consultations [23,24,25], while it increases access to services and cost-effectiveness [26,27,28].

In summary, interventions could facilitate communication and access to genetic information and services for families with hereditary predisposition to cancer. The purpose of this study was to identify and synthesize outcomes of psychoeducational interventions designed to facilitate family communication of genetic testing results and/or cancer predisposition cascade genetic testing, with a focus on HBOC and LS.

## 2. Materials and Methods

### 2.1. Literature Extraction

This systematic review is reported according to PRISMA (Preferred Reporting Items for Systematic Reviews and Meta-Analysis) guidelines [29]. The search strategy was designed to identify available randomized controlled trials (RCTs) that assessed the efficacy of interventions that included family communication of genetic testing results and/or cancer predisposition cascade genetic testing as a primary or a secondary outcome. Several criteria were used to select eligible studies: (1) the intervention had to involve mutation carriers, or blood relatives of known mutation carriers, or individuals with a strong family history indicative of HBOC or LS; (2) the intervention had to include a psychosocial, cognitive, or behavioral component; and (3) participants had to be randomly assigned to either the intervention or the control arm. The search strategies were developed by an information specialist (C.A.-H.) and peer-reviewed by a second information specialist (Dr. Hannah Ewald). The electronic databases Embase via Elsevier, Medline and PsycInfo via Ovid, and the Cochrane Central Register of Controlled Trials (CENTRAL) were searched using text word synonyms and database-specific subject headings for hereditary cancer, genetic counseling/screening, and interventions to promote family communication and/or genetic counseling/screening. For Embase, Medline, and PsycInfo, common RCT filters were applied [30,31] (last search June 15, 2020; Appendix A). References were exported to Endnote X9 [32] and de-duplicated using the Bramer method [33]. Queries were limited to studies published in the English language. Studies published in languages other than English were excluded due to time and resource limitations.

### 2.2. Screening, Inclusion, and Exclusion Criteria

Each research article was screened by title and abstract by at least two members of the research team (VB, MCK, MUB, and Dr. Tarsha Jones), who made an independent assessment among the full-text articles evaluated for eligibility. Disagreements were resolved through consensus. Papers with no original data, such as guidelines, study protocols, and reviews, were excluded. Only original articles assessing family communication and/or cancer predisposition cascade genetic testing for HBOC and LS were included. Studies involving patients with other types of cancer and/or other genetic conditions were excluded to reduce the heterogeneity of the studies analyzed. Full-text analysis was performed on 102 records selected during title and abstract screening.

### 2.3. Data Extraction

Data from eligible articles were extracted and were recorded using Covidence software [34]. We recorded the main author, year of publication, country of origin, study design, demographics of study population, and outcomes. Intervention content and components were also analyzed. In one case, specific intervention characteristics were obtained from an earlier publication that was identified from the reference list of the original article. When authors used more than one instrument to measure the same outcome, extracted data were reported from the most relevant instrument, which was determined by consensus after reviewing wording of each item. A similar procedure was followed when authors reported findings on multiple subscales of instruments, rather than on global scores. The Cochrane Risk of Bias (RoB) tool [35] was used to assess risk of bias in sequence generation (selection bias), allocation concealment (selection bias), blinding of participants and personnel (performance bias), blinding of outcome assessment (detection bias), incomplete outcome data (attrition bias), selective outcome reporting (reporting bias), and other potential sources. Based on the RoB tool, potential sources of bias were characterized as “low”, “high”, or “unclear” for each included study. Calculation of effect sizes was based on outcome data from the experimental and control arms of each study.

### 2.4. Statistical Analyses

Outcome data were synthesized using meta-analytic methods [36,37]. The standard mean difference, or the effect size between intervention and control groups, was calculated using Hedges’ *g* unbiased approach, which is similar to Cohen’s d statistic [38]. Calculation of effect sizes was based on means, standard deviations, difference in mean scores, odds ratios, *p*-values, and sample sizes of the groups. Data were statistically pooled by the standard meta-analytic approach, meaning that studies were weighted by the inverse of the sampling variance. For studies that did not report the coefficient of correlation (*r*) between pre- and post- intervention scores, we used Rosenthal’s conservative estimate of *r* = 0.7 [39]. The random effects model was used as a conservative approach to account for different sources of variation among studies. The Q statistic was used to assess heterogeneity among studies. A significant Q value indicates lack of homogeneity of findings among studies [36,37]. Due to the small number of studies, we were not able to conduct moderation analyses and examine the effects of intervention characteristics on outcomes. We assessed for publication bias using the Egger’s *t*-test with significance values based on one-tailed *p*-values [36,37]. Publication bias can occur because (i) journals are more likely to publish studies with positive results, (ii) authors are less likely to report negative or inconclusive outcomes in multi-outcome studies, or (iii) studies with smaller sample sizes need to detect larger effects to be published than studies with larger samples.

Comprehensive Meta-Analysis V.3© Software [40] was used for statistical analyses. Reported statistics conform to the PRISMA Statement [29]. Based on conventional standards, effect sizes of *g* = 0.20, 0.50, and 0.80 were considered small, medium, and large, respectively [38].

## 3. Results

**Figure 1:**
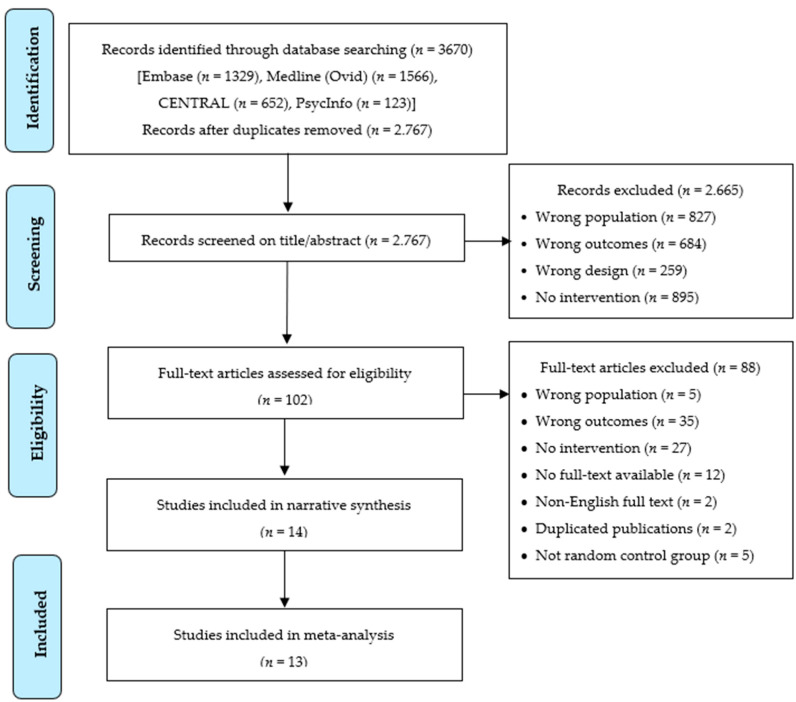
PRISMA flow diagram for study selection.

Initial queries identified 2767 articles from all databases and search methods after removing duplicates (see Figure 1 for details). We identified 14 studies that met all inclusion criteria and were included in the narrative synthesis. However, the meta-analysis was based on data extracted from 13 RCTs published between 2002 and July 2019 that assessed family communication and/or cascade genetic testing for HBOC and/or LS among outcomes. Outcomes from one RCT were not included in the calculation of pooled effect sizes due to missing data [41]. Studies measured outcomes at various time points, ranging from one week to 14 months post-intervention. The median time for post-intervention assessments was three months. When studies assessed outcomes multiple times, we used data from the time point closer to three months. For outcomes assessed only once, we used data from that time point. Quality assessment indicated that lack of participant blinding was the most common source of bias among included studies (Table S1).

### 3.1. Characteristics and Content of Interventions

Most interventions targeted HBOC (*n =* 5) [41,42,43,44,45], followed by interventions that targeted multiple hereditary syndromes, including both HBOC and LS (*n =* 4) [46,47,48,49], colorectal cancer associated with LS (*n =* 3) [50,51,52], or ovarian cancer (*n =* 2) [53,54]. Characteristics and content of the identified studies are described in Table 1. All protocols (*n =* 14) included a psychoeducational and/or counseling component; *n =* 5 included skills building; *n =* 4 included a decision aid; and *n =* 1 focused on motivational interviewing. The psychoeducational components focused on genetics and hereditary cancer risk, prevention and risk management options, and impact on family and/or family communication. The counseling component was designed to enhance coping, problem solving, self-efficacy, and clarifying personal values. Less often, protocols included resources for participants accessing genetic services (*n =* 3) or additional training and resources for clinicians to enhance referral for genetic counseling (*n =* 1). Most interventions were theoretically driven (*n =* 9); Buckman’s six-step strategy for “breaking bad news” was the most frequently mentioned theoretical approach, followed by the Ottawa Decision Support framework. Finally, most studies (*n =* 9) reported various outcomes related to intervention fidelity. Table 1 provides brief details about the content of controls and/or usual care of the identified RCTs.

### 3.2. Intervention Mode of Delivery and Intervener

Few protocols (*n =* 2) targeted implicitly or explicitly more than one member from the same family. Most interventions (*n =* 8) required extensive counseling sessions with a healthcare provider, often specified as a genetic counselor/geneticist, Master’s-prepared nurse, or psychosocial worker. Counseling involved mostly one-on-one sessions and was delivered entirely or partially over the telephone (*n =* 7). Interventions were developed either exclusively as booklets (*n =* 3) or included a paper handout as a complementary component (*n =* 4). Technology-enabled interventions were delivered either via the World Wide Web (*n =* 2), as a mobile app (*n =* 1), or included the audio recordings of the counseling sessions (*n =* 1).

### 3.3. Intervention Dose and Duration

The dose and duration of “received intervention” was not consistently reported among studies. Most protocols (*n =* 9) specified a dose of intervention that ranged from 1 to 7 contacts with participants, with an overall duration ranging from 20 to 140 min, over 7 days, 3 weeks, or 12 months.

### 3.4. Characteristics of Samples

Most studies were conducted in the US (*n =* 7), followed by Australia (*n =* 4), the Netherlands (*n =* 2), and Sweden (*n =* 1). Table 2 summarizes the sample characteristics of the 14 interventions included in the narrative synthesis. Sample sizes ranged from 24 to 490, with a total of 2968 participants across all studies. Recruitment in most studies (*n =* 8) was from outpatient settings. Enrollment rates varied from 23% to 96% of those approached, with an average enrollment of 71% across studies. Attrition ranged from 13% to 59%, with an average attrition of 27% across studies. Reasons for attrition were not consistently reported.

Most studies (*n =* 10) included over 50% female participants, the majority including 100% females (*n =* 7); few focused exclusively on ovarian cancer (*n =* 2); the remaining focused on HBOC (*n =* 5). A larger proportion of males was included in studies focusing on colorectal cancer and only one included a majority of male participants [50]. Race was not consistently reported, especially for studies conducted outside the US (*n =* 7). Studies that reported participants’ race included only or primarily White individuals, and only one included 59% Black individuals [46]. The reported mean ages ranged from 33 to 61. Participants were mostly well-educated among the studies that reported educational level (*n =* 7).

Most studies (*n =* 11) included both affected and unaffected individuals. Four studies reported whether participants had a pathogenic variant associated with cancer; all others focused on personal and/or family history of cancer to describe risk.

### 3.5. Effect Sizes Obtained for Outcomes

Table 3 presents an overview of meta-analytic findings for outcomes assessed. Family communication was the most commonly assessed outcome (*n =* 8), followed by knowledge (*n =* 7), cascade genetic testing (*n =* 6), anxiety (*n =* 4), depression (*n =* 4), and perceived risk (*n =* 3). Primary studies assessed additional outcomes, i.e., decisional conflict, decisional regret, coping, distress, and self-efficacy. However, calculation of pooled effect sizes for these additional outcomes was not possible, either because there were less than three studies or due to missing data. The table provides the pooled effect sizes for assessed outcomes, 95% confidence intervals, assessment of heterogeneity across studies (Q statistic), and Egger’s *t*-test for publication bias. Forest plots for each outcome are shown. Forest plots depict the effect sizes calculated for each study by outcome (■ symbol) as well as the overall effect size obtained for the outcome across studies (◆ symbol). Forest plots also indicate whether effects obtained in each study and across studies favor the control or the intervention.

**Figure 2:**
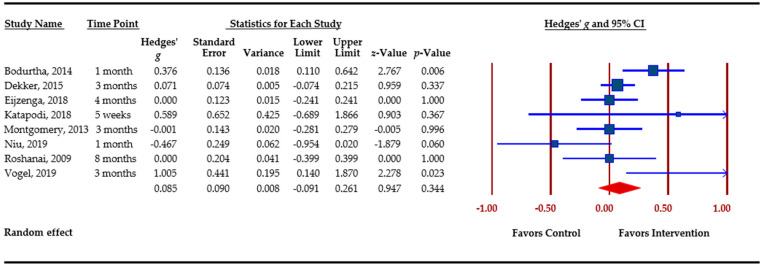
Family communication.

Family communication was conceptualized by primary studies most commonly as the number of relatives contacted/informed about the pathogenic variant, as well as frequency of contact and openness/ease of family communication. The Q statistic indicates significant heterogeneity among the eight studies that evaluated changes in family communication. The overall effect size was small and not significant, *g* = 0.085 (*p =* 0.344). (Figure 2). Among the eight studies, three assessed family communication as a secondary outcome [50,52,54]; removing these studies did not change the significance of the pooled effect size.

**Figure 3:**
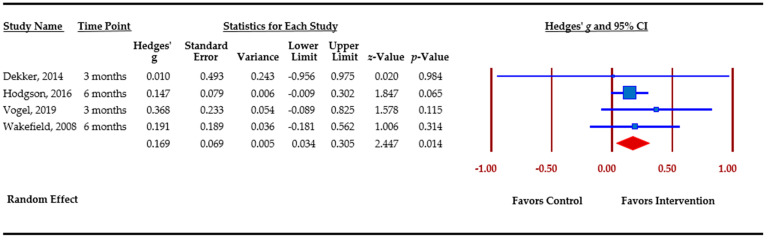
Cascade genetic testing.

Cascade genetic testing was assessed by six primary studies as uptake of genetic testing by relatives and/or contact with genetic services and request for genetic consultation. The assessment was based on participants’ self-reports, and/or less often on clinic records. The overall effect size was small and not significant, *g* = 0.086 (−0.075–0.247) (*p* = 0.295). However, two of these studies [51,53] assessed cascade genetic testing as a secondary outcome. Removing these two studies changed the overall effect size, which remained small but significant, *g* = 0.169 (*p =* 0.014). (Figure 3). Effect sizes among primary studies ranged from 0.010 to 0.368.

**Figure 4:**
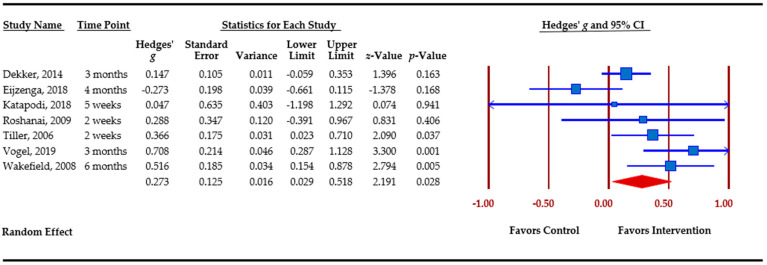
Knowledge.

Knowledge was conceptualized by primary studies as knowledge of heredity and cancer genetics, knowledge of risk factors and familial risk, and knowledge related to genetic testing. The Q statistic indicates significant heterogeneity among the seven studies that evaluated changes in knowledge. The overall effect size was small but significant, *g* = 0.244 (*p <* 0.001), favoring the intervention arm. Effect sizes among primary studies varied between −0.273 and 0.708 (Figure 4).

**Figure 5:**
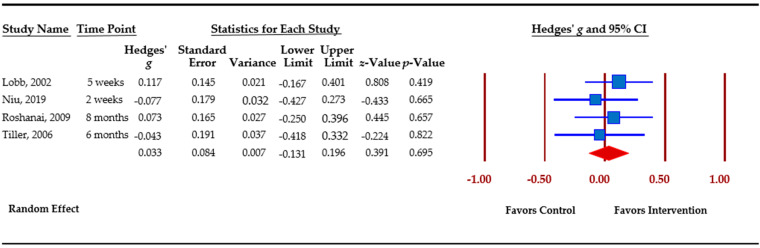
Anxiety.

Anxiety was assessed by primary studies with the Hospital Anxiety and Depression Scale (HADS) [55] and the Spielberger State Trait Anxiety Inventory (STAI) [56]. Egger’s *t*-test indicates publication bias for the four studies that evaluated changes in anxiety. The overall effect size was small and not significant, *g* = 0.033 (*p =* 0.695). (Figure 5).

**Figure 6:**
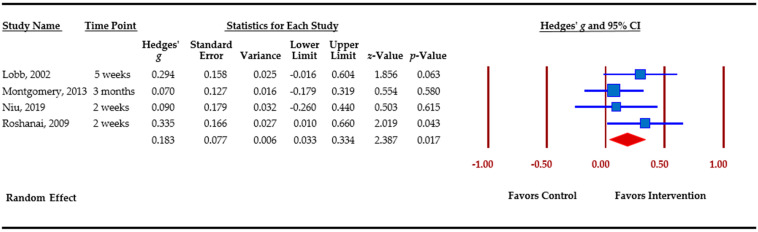
Depression.

Depression was assessed by primary studies using the Hospital Anxiety and Depression Scale (HADS) [55] and the Centers for Epidemiological Studies—Depression scale (CESD) [57]. Among the four studies that evaluated changes in depression, the overall effect size was small but significant, *g* = 0.183 (*p =* 0.017), favoring the intervention arm. Effect sizes among individual studies varied between 0.070 and 0.335 (Figure 6).

**Figure 7:**
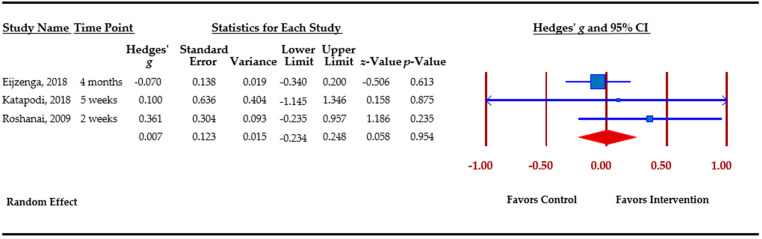
Perceived risk.

Perceived risk for developing cancer was assessed in three studies. Changes in perceived risk were small and not significant, *g* = 0.007 (*p* = 0.95). (Figure 7).

## 4. Discussion

The primary purpose of this paper was to identify interventions that were designed to facilitate family communication of cancer genetic testing results and/or cascade genetic testing among blood relatives, with a focus on HBOC and LS. To enhance the methodological rigor of our review, we focused exclusively on studies that tested intervention efficacy with an RCT design. Our systematic search identified 14 studies that met all inclusion criteria and were included in the narrative synthesis of this paper about intervention components, mode of delivery, and sample characteristics. Meta-analysis of outcomes was possible only for 13 studies, and not all of them had assessed family communication of test results and/or cascade genetic testing of relatives as a primary outcome. Our literature search identified serendipitously additional papers describing the development of relevant interventions [58,59,60,61,62,63,64,65]. However, none of them had been rigorously tested with an RCT, indicating that the scientific field is still in development. Our findings indicate that this is a growing field with significant heterogeneity of approaches, with few rigorously tested interventions that genetic professionals can emulate in cancer genetic practices.

The 14 identified interventions delivered to carriers of pathogenic variants and/or their blood relatives were comprehensive and addressed family communication, cascade testing of relatives, knowledge of cancer genetics, and psychosocial wellbeing as primary or secondary outcomes. We recorded three indicators of intervention quality. First, most studies included theory-driven intervention protocols, which decreases the likelihood of isolated or chance findings. However, there was considerable variability, with some studies mentioning the theory in passing or in generic terms, while others indicated specific theories and demonstrated how the theory was utilized in the selection of intervention content and choice of outcomes. Second, fewer studies instituted ways to examine intervention fidelity, i.e., the extent to which the protocol was delivered in a consistent manner. Investigators used protocol manuals, intervention logs, and/or tape-recorded sessions to assess or maintain intervention fidelity, indicating a growing understanding of the importance of adherence to standardized protocols. Third, there was considerable variability in intervention “dose” among protocols, both in the number of sessions (range 1 to 7 contacts) and duration of interventions (ranging from 20 to 140 min, delivered over 7 days to 12 months). Detailed information about intervention dose was not consistently reported. Intervention dose could be further evaluated or standardized within studies; otherwise, it is difficult to determine if, or how much of, the intervention “dose” affects outcomes.

The majority of protocols included one-on-one and face-to-face or telephone extensive counseling with a trained healthcare provider, often identified as a genetic counselor or Master’s-prepared nurse. Moreover, few interventions were delivered via a web-based or mobile app platform. Given the shortfall of trained genetic health professionals, technology-based approaches are needed to extend the reach to individuals weighing genetic testing decisions and facilitating cascade genetic testing. Increased access to genetic information could be facilitated with web-based or mobile health technologies. The availability of internet access, rising levels of electronic literacy, and the growing number of patient portals/web-based approaches hold promise for expanding the reach of tailored, cost-effective genetic care [27,66]. Technology-enabled education and tele-genetics is equivalent to face-to-face consultations in presenting the benefits and drawbacks of genetic testing at half the cost of traditional consultations [24,66].

Content related to the implications of genetic test results for blood relatives and communication was included in most interventions. However, the overall effect size for this outcome was small and not significant. There was significant heterogeneity among protocols, ranging from booklets that carriers could pass on to untested relatives, to family-based communication training. Some studies assessed communication as a secondary outcome. Taken together, these findings suggest that although building communication skills and/or providing support for dissemination of genetic testing results is an essential component, little is known about the best approach to enhance this outcome [67]. From the 14 protocols included in the narrative synthesis, many included extensive meetings with a healthcare provider, suggesting some individualization and tailoring of intervention content. However, most protocols targeted only carriers’ communication skills and coping strategies, who are the transmitters of genetic information, and did not address communication and coping of relatives, who are the recipients. Communication of genetic test results is a two-way exchange between carriers and relatives and should be addressed as a family-based outcome, yet only two protocols included both a carrier and untested relatives. Enhancing communication of genetic testing results should be guided by family-based theoretical frameworks and tested with family-based designs [68,69,70].

When cascade genetic testing was the primary focus of interventions, the overall effect was small but significant. However, this finding should be interpreted with caution due to the small number of studies and the outcome based on self-reports. Invitation letters for genetic counseling, list of genetic resources, repeated contact with carriers over 12 months, and enhancing physician referrals were some of the techniques employed by the reviewed interventions. The current legal framework does not support healthcare professionals directly contacting blood relatives. However, removing this barrier does not guarantee successful cascading of blood relatives due to the resources needed to identify, contact, and counsel them. Additional measures, such as mailing of saliva kits [64] and family-based telephone or web-based counseling, hold promise to enhance cascade genetic testing and improve individual and population health outcomes.

Content related to cancer genetics, modes of inheritance, and risk factors was included in all interventions, resulting in a small but significant overall effect size and suggesting that this is an essential content area. This finding is consistent with an earlier review reporting that risk communication during genetic consultations increases genetic knowledge [71]. The significant heterogeneity observed for this outcome could be due to the different measures used to assess knowledge of cancer genetics, or due to the different syndromes and/or cancer types (e.g., colorectal or ovarian cancer) that were the focus of each intervention. Moreover, there is significant heterogeneity among counselees’ preferences, with some preferring to receive detailed genetic information while others preferring “just the basics” [72,73], making streamlining lay genetic education difficult without a tailored approach.

Psychosocial outcomes, such as anxiety, depression, and perceived risk, as well as decisional conflict, regret, coping, and satisfaction were not assessed consistently among studies. Thus, we were unable to calculate pooled effect sizes for many of these outcomes. A significant number of interventions included decision aids, exercises for value clarification, and provided information on preventive and risk management options. These components likely enhance psychosocial adjustment to hereditary cancer risk and increase emotional wellbeing [71]. Although primary studies used validated instruments to assess these outcomes, meta-analysis findings regarding intervention efficacy, heterogeneity, and publication bias should be interpreted with caution due to the small number of primary studies and the heterogeneity of syndromes and/or cancer types. Risk communication in the clinical context resulted in general improvement for these outcomes [71].

Little is known about samples of racially, ethnically, and social diverse backgrounds. Only one study included a majority of Black participants, and only one study included a majority of male participants, indicating significant knowledge gaps regarding family communication and cascade genetic testing in males, especially in the context of HBOC. Future studies should also focus on LS, as it is the most common hereditary cancer condition known today, but remains largely undetected due to the different cancer types associated with LS and the lack of clear diagnostic criteria [74,75,76,77].

## 5. Limitations

We did not include studies published in languages other than English, unpublished studies, and abstracts from conference proceedings to ensure that findings were based on higher-quality, peer-reviewed studies. Excluding unpublished studies is likely to introduce an upward bias into the size of the effects found, which means that calculated effect sizes are likely to be larger [37]. To address this limitation, we assessed the heterogeneity of findings with the Q statistic and publication bias with the Egger’s *t*-test statistic. Publication bias appeared only in one outcome and may be related to the small number of studies. Our findings are comparable to a previous review assessing psychosocial outcomes of genetic counseling [62]. Finally, due to the small number of studies and the diverse outcomes, we were not able to conduct moderation analyses and examine the impact of similar types of interventions on outcomes (e.g., web-based vs. paper-based). The heterogeneity and attrition across studies also decrease our ability to discern the clinical utility of these interventions.

The time span of studies included in our meta-analysis covered a period of 17 years, during which there have been massive shifts in clinical practice and in public understanding of genetic testing. The introduction of panel testing has created new complexities in managing hereditary cancer risk associated with pathogenic variants of moderate penetrance, which may further contribute to existing barriers to family communication and cascade testing. GINA (Genetic Information Non-Discrimination Act), which was passed in the US in 2008 [78], may have lessened concerns about genetic discrimination, facilitating family communication and cascade genetic testing. However, this applies only to the seven studies conducted in the US, while the legal framework for protecting genetic information in other countries is not known. Discerning the influence of these two factors on family communication and cascade genetic testing is not possible under the scope of this study.

## 6. Conclusions

At the time of conducting this study, no similar reviews about family communication and/or cascade genetic testing for hereditary cancer syndromes have been published. Research has been mainly focused on helping healthcare professionals to facilitate family communication about genetic test results, and uptake of cascade testing has increased due to educational materials and technological resources and due to the active involvement of healthcare providers [79].

Although professional organizations call for the implementation of cascade testing for HBOC and LS, debate remains about the conflict between the need to protect the privacy of tested individuals and the rights of blood relatives to be notified about genetic information. Facilitating this process will contribute to the implementation of cascade genetic testing and significantly reduce the burden of cancer resulting from familial pathogenic variants. Technology- and theory-driven, rigorously-tested, psychoeducational interventions could play a significant role in this public health effort. Our study highlights the need for developing new interventions and new approaches in family communication and cascade testing for cancer susceptibility, laying the foundation for future work to address current knowledge gaps. Future studies could compare interventions assessing these outcomes regardless of the genetic condition, assuming similar “actionability” of genetic findings. Rigorous testing of promising interventions using an RCT design will propel the scientific field forward. In addition to individual- and family-level interventions, consideration should be given to health system and policy-level changes that might facilitate the communication of cancer genetic risk information and cascade testing.

## Figures and Tables

**Table 1:** Characteristics of study interventions.

Author/Year	Syndrome/Outcomes *	Intervention	Control	Theoretical Framework	Mode of Delivery	Intervener	Dose	Duration	Fidelity
Bodurtha et al., 2014 [46], KinFact	Both/Communication	Booklet (27-page personalized information for family communication about cancer and cancer genetics)	Pamphlet—breast, colon cancer risks, screening, services	Health Belief Model; Buckman’s 6-step strategy Breaking Bad News	Booklet/PamphletFace-to-face One-on-one	Trained Personnel	Once	20-min	NR~
Dekker et al., 2015 [50]	CRC **/Communication Cascade testing Knowledge	Website (CRC risk, risk calculators, decision aid) + Brochure (familial CRC risk, prevention) + 30-min Clinician education + Referral cards (criteria)	Usual care	NR	Website + Brochure	Self-administered	NR	NR	67% used website
Eijzenga et al., 2018 [47]	Both/Communication Knowledge Perceived risk	Standard genetic counseling + Phone call—motivational interviewing (enhance family communication, knowledge, motivation, self-efficacy, solutions)	Standard genetic counseling	Motivational interviewing	Telephone	Psychosocial Worker	Once	NR	33% random check interview recording
Hodgson et al., 2016 [48]	Multiple incl. HBOC + LSCascade testing	Enhanced genetic counseling over telephone with emphasis on family communication + Pedigree	Pedigree	NR	Telephone One-on-one	Genetic Counselors	2–3 times	12 months	NR
Katapodi et al., 2018 [42] Family Gene Toolkit	HBOC/CommunicationKnowledge Perceived risk	Webinar (power point, live presentations about cancer genetics, risk, genetic counseling, coping, family communication) + Decision aid + Communication skills building + Phone call	Wait-listed control	Theory of Stress and Coping	Web-based + Telephone Face-to-face One-on-family + One-on-one	Genetic Counselor + Master’s Oncology Nurse	2 webinars 45–60 min per webinar + 20 min phone call	3 weeks 110–140 min	71% completion rate
Loader et al., 2002 [51]	CRC/Cascade testing	Brochure (hereditary cancer, risk factors, prevention, genetic testing, family communication) + Invitation to counseling + Letter genetic counseling	Physician education (CRC risk, information about referrals to counseling)	NR	Brochure + Letter Face-to-face One-on-one	Mail, Self-administered	Once	NR	47% counseled
Lobb et al., 2002 [43]	HBOC/Anxiety Depression	Audio-recording of genetic consultation	Usual care	NR	Audiotapes	Self-administered	NR	NR	51% listened tape once
McInerney-Leo et al., 2004 *** [41]	HBOC	Family education + Problem Solving Training (expectations, concerns, feelings) for task- and emotional-focused coping and problem solving + Telephone interview	Family education + Client-centered counseling + Telephone interview	Cognitive–Behavioral Theory	Face-to-face or TelephoneOne-on-family + One-on-one	Trained Provider	Once	60 min	Standardized protocol
Montgomery et al., 2013 [44]	HBOC/Communication Depression	Counseling (risk factors, personal risk, pedigree) + Communication skills building (who, how, extent willing to know, share results, emotional responses, resources)	Wellness education (nutrition, exercise) + List of nutrition websites	Buckman’s 6-step strategy Breaking Bad News + Theory of Planned Behavior	Face-to-face One-on-one	Genetic Counselor + Research Staff	NR	NR	NR
Niu et al., 2019 [52]	CRC/Communication Anxiety Depression	Genetic counseling + Clinical exome sequencing (21 to >50 actionable genes) + Additional genetic information	Counseling + Tumor testing OR panel testing + Review family history	NR	Telephone or Face-to-faceOne-on-one	Genetic Counselor or Geneticist	NR	NR	NR
Roshanai et al., 2009 [49]	Both/Communication Knowledge Anxiety DepressionPerceived risk	Genetic counseling + Extended meeting nurse specialist (pedigree, cancer risk, 6-step strategy for family communication) + Pamphlet + Videotape of counseling + Copies pedigree, medical records	Genetic counseling + Short meeting nurse specialist (intention inform relatives) + Videotape of counseling	Buckman’s 6-step strategy Breaking Bad News	Clinical settingFace-to-face One-on-one	Genetic Counselor + Nurse Specialist	Once	NR	19-item survey counselees
Tiller et al., 2006 [53]	Ovarian Cancer/Knowledge Anxiety	Decision aid (booklet on risk factors, family history and risk, genetic testing, prevention) + Values clarification	General education pamphlet	Ottawa Decision Support Framework	Pamphlet	Self-administered	Once	NR	88% review booklet
Vogel et al., 2019 [54]mAGIC	Ovarian cancer/Communication Cascade testing Knowledge	Mobile app tailored messages (motivation, positive feedback, triggers) + Videos (genetic counseling, testing, personal health, cancer genetics, self-care, self-efficacy) + Training how to use mAGIC + Pamphlet (ovarian cancer risk, counseling, services)	Usual care + Pamphlet (hereditary cancer risk, counseling, services)	Health Belief Model + Fogg Behavioral Model of Mobile Persuasion	Mobile app + Pamphlet	Self-administered	Once per day10–15 min per day	7 days70–90 min	NR
Wakefield et al., 2008 [45]	HBOC/Cascade testing Knowledge	Decision aid (40-page booklet, hereditary cancer, testing, impact on individual and family) + Values clarification	Pamphlet (4-page education about HBOC genetic testing)	Ottawa Decision Support Framework	Brochure/Pamphlet	Self-administered	NR	NR	70% intervention read booklet

* Study outcomes included in the meta-analysis. Individual studies may have assessed additional outcomes that were not included because it was not possible to calculate effect sizes. ** CRC, Colorectal cancer. *** Intervention is not included in calculation of effect sizes due to missing data. ~ NR = Not Reported.

**Table 2:** Sample characteristics.

Author/Year Country	Setting	Sample N	Cancer Type/Stage/PDx *	Carrier of PV ** or FH ***	Age Mean ± SD or Range	Sex	Race	Education% ≤ HS^	Enrollment	Attrition
Bodurtha et., al 2014 [46], USA	Outpatient	490	Stage/type NR: HBOC or CRC risk	75% FDR^+^ any cancer 10% FH breast or CRC	33.4 ± 11.9	100% female	59% Black 33% White 8% Other/Multiple	41% 16% missing	61%	42%
Dekker et al., 2015 [50], Netherlands	Hospital	384	100% CRC I: 86.4% Stage I–III C: 86.55 Stage I–III	I: 9% high risk C:13% high risk	I: 60 ± 8.2 C: 59 ± 7.5	I: 71% male C: 66% male	NR~	NR	55%	59%
Eijzenga et al., 2018 [47], Netherlands	Hospital	305	Stage/type NR; HBOC or CRC risk I: 70% PDx C: 73% PDx	I: 9% PV C: 12% PV	I: 53.1 ± 10.1 C: 54.4 ± 12.4	I: 75% female C:75% female	NR	I: 36%C: 30%	90%	21%
Hodgson et al., 2016 [48], Australia	Hospital and Genetic Clinic	95	Stage/type NR; HBOC and LS	I: 57.8% “actionable” groupC: 50.0% “actionable” group	I: 49.5 ± 14.9 C: 45.8 ± 13.9	I: 50% female C:48% female	NR	NR	57%	53%
Katapodi et al., 2018 [42], USA	Outpatient	24	Stage/type NR: HBOC 40% PDx Breast 10% PDx Ovarian 20% PDx Other	12 PV	41 ± 13	100% female	100% White	NR	23%	29%
Loader et al., 2002 [51], USA	Cancer Registry	101	100% PDxCRC; stage NR	100% ≥1 FDR or SDR^++^ CRC	Not Counseled: 57.3 ± 6.9 Counseled:59.2 ± 6.5	53% female	93% White	NR	71%	13%
Lobb et al., 2002 [43], Australia	Outpatient	193	Stage/type NR; HBOC I: 42% PDx C: 45% PDx	NR	I: 45 C: 44	100% female	NR	I: 47% C: 50%	88%	18%
McInerney-Leo et al., 2004 [41], USA	NR	262	Stage/type NR; HBOC families	26% PV 85% genetic testing	55% ≥ 40	65% female	Mostly White	NR	47%	19%
Montgomery et al., 2013 [44], USA	Outpatient	422	Stage/type NR; HBOC	NR	48.5 ± 11.0	100% female	95% White	77%	96%	41%
Niu et al., 2019 [52], USA	Outpatient	190	I: 33.68% CRC PDx C: 36.84% CRC PDx	NR	I: 53.4 ± 12.5 C: 51.8 ± 14.0	I: 46% female C:57% female	I: 81% White C: 84% White	NR	NR	26%
Roshanai et al., 2009 [49], Sweden	Outpatient	147	HBOC, CRC riskI: 38.36% PDxC: 35.14% PDx	I: 77% No PDx >20%risk 79% PDx >20% risk C:81% No PDx >20% risk 70% PDx >20% risk	56 (23-84)	I: 92% female C: 89% female	NR	NR	66%	15%
Tiller et al., 2006 [53], Australia	Outpatient	131	Ovarian cancer I: 51.5% PDx C: 52.4% PDx	I: 74.2% FH C: 71.4% FH	I: 45.8 C: 46.3	100% female	NR	I: 29% C:29%	92%	17%
Vogel et al., 2019 [54], USA	Outpatient	104	Ovarian cancer 100% PDx I: ≥74% Stage III C: ≥75% Stage III	NR	I: 60.9 ± 10.7 C: 61 ± 12	100% female	I: 91% White C: 88% White	I: 20.8% C: 18%	82%	13%
Wakefield et., al 2008 [45], Australia	Outpatient	120	Type NR; HBOC I:56.1% PDx C:65.1% PDx	100% FH HBOC—cancer	I: 45.8 (21–73) C: 49.6 (22–75)	100% female	NR	I: 26.3% C: 36.5%	94%	17%

* PDx = Personal cancer diagnosis. ** PV = Pathogenic variant. *** FH = Family history of cancer. ^ %≤HS = Percentage of participants with education equal or less than high school. +FDR = First-degree relatives. ++SDR = Second-degree relatives. ~ NR = Not Reported.

**Table 3:** Pooled effect sizes of outcomes.

Outcomes	Number of Trials	Overall Sample N	Pooled Effect Size Hedges’ *g* (95% CI)	Q for Heterogeneity	Egger’s *t*-Test for Publication Bias
Family communication	8	2066	0.085 (−0.091 – 0.261)	15.50*	0.53
Cascade genetic testing	4	703	0.169 (0.034 – 0.305)*	0.93	−0.66
Knowledge	7	1215	0.244 (0.109 – 0.379)*	15.10 *	0.50
Anxiety	4	661	0.033 (−0.132 – 0.198)	6.14	−4.17*
Depression	4	952	0.183 (0.033 – 0.334)*	2.39	2.89
Risk perception	3	476	0.007 (−0.230 – 0.250)	1.69	0.97

* *p*-value ≤ 0.05.

## Supplementary Materials

The following are available online at https://www.mdpi.com/2072-6694/13/4/925/s1, Table S1. Tabular representation of risk of bias in individual studies.

## Appendix A.

### Search Strategies

Embase.com

(20200615; 1,329 hits)

(‘hereditary breast and ovarian cancer syndrome’/de OR ‘hereditary nonpolyposis colorectal cancer’/de OR ‘BRCA1 protein’/de OR ‘BRCA2 protein’/de OR ‘BRCA1 protein human’/de OR ‘BRCA2 protein human’/de OR ‘BRCA gene’/de OR ‘BRCA2 gene’/de OR ‘BRCA protein’/de OR ‘MutL protein homolog 1′/de OR ‘mlh1 gene’/de OR ‘mlh1 protein human’/de OR ‘DNA mismatch repair protein MSH2′/de OR ‘msh2 gene’/de OR ‘msh2 protein human’/de OR ‘protein MSH6′/de OR ‘mismatch repair protein PMS2′/de OR ‘pms2 gene’/de OR ‘pms2 protein human’/de OR ‘epithelial cell adhesion molecule’/de OR ‘epcam gene’/de OR ‘epcam protein human’/de OR (‘HBOC syndrome’ OR ((‘hereditary nonpolyposis’ OR ‘hereditary non polyposis’) NEXT/3 (cancer OR neoplasm*)) OR HNPCC OR ‘Lynch syndrome’ OR ‘Lynch II syndrome’ OR ‘Muir Torre syndrome’ OR BRCA* OR FANCD* OR (‘Fanconi anaemia’ NEXT/3 D1) OR (MutL NEXT/2 ‘homolog 1′) OR MLH1 OR hMLH1 OR ‘MutS homolog 2′ OR MSH2 OR hMSH2 OR MSH6 OR ‘postmeiotic segregation increased protein 2′ OR PMS2 OR ‘epithelial cell adhesion molecule’ OR EPCAM):ab,ti OR ‘hereditary tumor syndrome’/de OR ‘cancer risk’/de OR ‘cancer susceptibility’/de OR ‘oncogene’/de OR ‘tumor suppressor gene’/de OR ‘tumor gene’/de OR ‘cancer genetics’/de OR (oncogene OR ((cancer* OR tumor* OR tumour*) NEAR/3 (syndrome OR risk OR predisposition OR susceptibility OR anticipation OR prognosis OR disorder OR gene*))):ab,ti OR ((‘genetic predisposition’/exp OR ‘genetic risk’/de OR ‘gene mutation’/de OR ‘genetic disorder’/de OR ‘single nucleotide polymorphism’/de OR ‘family history’/de OR (hereditary OR inherit* OR inborn OR familial OR mutation* OR genetic* OR ((family OR genomic*) NEAR/3 (syndrome OR risk OR predisposition OR disposition OR susceptibility OR anticipation OR prognosis OR disorder OR condition* OR history)) OR ‘single nucleotide polymorphism*’ OR SNP OR SNPs):ab,ti) AND (‘neoplasm’/exp OR (cancer* OR neoplas* OR tumor* OR tumour* OR carcinoma* OR carcinogenesis OR malignan*):ab,ti)))

and

(‘counseling’/de OR ‘genetic counseling’/de OR ‘patient counseling’/de OR ‘family counseling’/de OR ‘consultation’/exp OR ‘decision support system’/exp OR ‘decision aid’/de OR ‘interpersonal communication’/de OR ‘persuasive communication’/de OR ‘patient information’/de OR ‘medical information’/de OR ‘health education’/de OR ‘patient education’/de OR ‘education program’/de OR ‘mass communication’/exp OR ‘telephone interview’/de OR ‘online system’/de OR ‘questionnaire’/exp OR ‘computer’/de OR ‘compact disk’/exp OR ‘mobile application’/exp OR ‘website’/de OR ‘multimedia’/de OR ‘digital health’/de OR ‘digital health technology’/de OR ‘digital health intervention’/de OR ‘telehealth’/exp OR ‘mhealth’/de OR ‘psychosocial care’/de OR ‘social support’/de OR (((family OR genetic OR genomic OR patient* OR intervention OR risk*) NEAR/3 counsel*) OR ‘preventive genetics’ OR consultation* OR teleconsultation* OR (decision NEAR/2 (support* OR aid* OR framework OR computer-assisted)) OR communicat* OR disclos* OR persuasion OR ((medical OR health OR patient* OR cancer OR risk* OR program*) NEAR/3 (information OR education)) OR internet OR ‘world wide web’ OR web-based OR online OR ‘social media’ OR facebook OR twitter OR ((cell OR cellular OR mobile OR smart) NEXT phone*) OR cellphone* OR smartphone* OR mail OR (postal NEXT (delivery OR service)) OR letter* OR telehealth OR tele-health OR ehealth OR e-health OR ‘digital health’ OR mhealth OR telemedicine OR tele-medicine OR telephone* OR dataphone* OR videoconferenc* OR ‘video conferenc*’ OR webcast OR computer* OR questionnaire* OR survey* OR ‘compact disk’ OR ‘CD-I’ OR ‘CD-ROM’ OR DVD OR ((mobile OR portable OR educational) NEXT/2 (app OR apps OR application*)) OR website* OR ‘web site*’ OR multimedia OR ((psychosocial OR social) NEXT (care OR support OR therapy OR intervention*)) OR telegenetic OR ‘educational material*’ OR ‘tailored message*’ OR ‘message tailoring’):ab,ti)

and

(‘genetic service’/exp OR ‘genetic analysis’/de OR ‘mutational analysis’/exp OR ‘DNA sequencing’/de OR ‘genetic discrimination’/de OR ‘genetic diagnosis’/de OR ‘family therapy’/de OR ‘family counseling’/de OR ‘family study’/de OR ‘informed decision making’/de OR ‘informed choice’/de OR (((family OR genetic* OR genomic* OR cascade OR mutation*) NEAR/3 (counsel* OR care OR testing OR screening OR analys* OR study OR studies OR discrimination OR diagnos*)) OR ‘DNA sequencing’ OR ‘preventive genetics’ OR ((family OR relative*) NEAR/3 (communicat* OR intervention* OR inform*)) OR (informed NEXT (decision* OR choice*))):ab,ti)

and

(‘crossover procedure’:de OR ‘double-blind procedure’:de OR ‘randomized controlled trial’:de OR ‘single-blind procedure’:de OR (random* OR factorial* OR crossover* OR cross NEXT/1 over* OR placebo* OR doubl* NEAR/1 blind* OR singl* NEAR/1 blind* OR assign* OR allocat* OR volunteer*):de,ab,ti)

Medline (Ovid)

(20200615; 1,566 hits)

(“hereditary breast and ovarian cancer syndrome”/OR Colorectal Neoplasms, Hereditary Nonpolyposis/OR BRCA1 protein/OR BRCA1 protein, human.nm. OR BRCA2 protein/OR BRCA2 protein, human.nm. OR Genes, BRCA1/OR Genes, BRCA2/OR exp MutL proteins/OR MLH1 protein, human.nm. OR G-T mismatch-binding protein.nm. OR MSH2 protein, human.nm. OR exp MutS proteins/OR PMS2 protein, human.nm. OR epithelial cell adhesion molecule/OR EPCAM protein, human.nm. OR (HBOC syndrome OR ((hereditary nonpolyposis OR hereditary non polyposis) ADJ3 (cancer OR neoplasm*)) OR HNPCC OR Lynch syndrome OR Lynch II syndrome OR Muir Torre syndrome OR BRCA* OR FANCD* OR (Fanconi anaemia ADJ3 D1) OR (MutL ADJ2 homolog 1) OR MLH1 OR hMLH1 OR MutS homolog 2 OR MSH2 OR hMSH2 OR MSH6 OR postmeiotic segregation increased protein 2 OR PMS2 OR epithelial cell adhesion molecule OR EPCAM).ab,ti. OR Neoplastic Syndromes, Hereditary/OR oncogenes/OR Genes, Tumor Suppressor/OR Genes, Neoplasm/OR (oncogene OR ((cancer* OR tumor* OR tumour*) ADJ3 (syndrome OR risk OR predisposition OR susceptibility OR anticipation OR prognosis OR disorder OR gene*))).ab,ti. OR ((exp Genetic Predisposition to Disease/OR mutation/OR Genetic Diseases, Inborn/OR Polymorphism, Single Nucleotide/OR (hereditary OR inherit* OR inborn OR familial OR mutation* OR genetic* OR ((family OR genomic*) ADJ3 (syndrome OR risk OR predisposition OR disposition OR susceptibility OR anticipation OR prognosis OR disorder OR condition* OR history)) OR single nucleotide polymorphism* OR SNP OR SNPs).ab,ti.) AND (exp neoplasms/OR (cancer* OR neoplas* OR tumor* OR tumour* OR carcinoma* OR carcinogenesis OR malignan*).ab,ti.)))

and

(counseling/OR genetic counseling/OR Referral and Consultation/OR exp Remote Consultation/OR decision support systems, clinical/OR Decision Support Techniques/OR communication/OR persuasive communication/OR health education/OR patient education as topic/OR exp telecommunications/OR Interviews as Topic/OR online systems/OR “Surveys and Questionnaires”/OR computers/OR exp compact disks/OR mobile applications/OR multimedia/OR telemedicine/OR exp social support/OR (((family OR genetic OR genomic OR patient* OR intervention OR risk*) ADJ3 counsel*) OR preventive genetics OR consultation* OR teleconsultation* OR (decision ADJ2 (support* OR aid* OR framework OR computer-assisted)) OR communicat* OR disclos* OR persuasion OR ((medical OR health OR patient* OR cancer OR risk* OR program*) ADJ3 (information OR education)) OR internet OR world wide web OR web-based OR online OR social media OR facebook OR twitter OR ((cell OR cellular OR mobile OR smart) ADJ phone*) OR cellphone* OR smartphone* OR mail OR (postal ADJ (delivery OR service)) OR letter* OR telehealth OR tele-health OR ehealth OR e-health OR digital health OR mhealth OR telemedicine OR tele-medicine OR telephone* OR dataphone* OR videoconferenc* OR video conferenc* OR webcast OR computer* OR questionnaire* OR survey* OR compact disk OR CD-I OR CD-ROM OR DVD OR ((mobile OR portable OR educational) ADJ2 (app OR apps OR application*)) OR website* OR web site* OR multimedia OR ((psychosocial OR social) ADJ (care OR support OR therapy OR intervention*)) OR telegenetic OR educational material* OR tailored message* OR message tailoring).ab,ti.)

and

(exp genetic services/OR DNA mutational analysis/OR Sequence Analysis, DNA/OR family therapy/OR (((family OR genetic* OR genomic* OR cascade OR mutation*) ADJ3 (counsel* OR care OR testing OR screening OR analys* OR study OR studies OR discrimination OR diagnos*)) OR DNA sequencing OR preventive genetics OR ((family OR relative*) ADJ3 (communicat* OR intervention* OR inform*)) OR (informed ADJ (decision* OR choice*))).ab,ti.)

and

(randomized controlled trial.pt. OR controlled clinical trial.pt. OR randomized.ti,ab. OR placebo.ti,ab. OR drug therapy.fs. OR randomly.ti,ab. OR trial.ti,ab. OR groups.ti,ab. NOT (exp animals/NOT exp humans/))

CENTRAL

(20200615; 652 hits)

((‘HBOC syndrome’ OR ((‘hereditary nonpolyposis’ OR ‘hereditary non polyposis’) NEXT/3 (cancer OR neoplasm*)) OR HNPCC OR ‘Lynch syndrome’ OR ‘Lynch II syndrome’ OR ‘Muir Torre syndrome’ OR BRCA* OR FANCD* OR (‘Fanconi anaemia’ NEXT/3 D1) OR (MutL NEXT/2 ‘homolog 1′) OR MLH1 OR hMLH1 OR ‘MutS homolog 2′ OR MSH2 OR hMSH2 OR MSH6 OR ‘postmeiotic segregation increased protein 2′ OR PMS2 OR ‘epithelial cell adhesion molecule’ OR EPCAM):ab,ti OR (oncogene OR ((cancer* OR tumor* OR tumour*) NEAR/3 (syndrome OR risk OR predisposition OR susceptibility OR anticipation OR prognosis OR disorder OR gene*))):ab,ti OR (((hereditary OR inherit* OR inborn OR familial OR mutation* OR genetic* OR ((family OR genomic*) NEAR/3 (syndrome OR risk OR predisposition OR disposition OR susceptibility OR anticipation OR prognosis OR disorder OR condition* OR history)) OR ‘single nucleotide polymorphism*’ OR SNP OR SNPs):ab,ti) AND ((cancer* OR neoplas* OR tumor* OR tumour* OR carcinoma* OR carcinogenesis OR malignan*):ab,ti))) AND ((((family OR genetic OR genomic OR patient* OR intervention OR risk*) NEAR/3 counsel*) OR ‘preventive genetics’ OR consultation* OR teleconsultation* OR (decision NEAR/2 (support* OR aid* OR framework OR computer-assisted)) OR communicat* OR disclos* OR persuasion OR ((medical OR health OR patient* OR cancer OR risk* OR program*) NEAR/3 (information OR education)) OR internet OR ‘world wide web’ OR web-based OR online OR ‘social media’ OR facebook OR twitter OR ((cell OR cellular OR mobile OR smart) NEXT phone*) OR cellphone* OR smartphone* OR mail OR (postal NEXT (delivery OR service)) OR letter* OR telehealth OR tele-health OR ehealth OR e-health OR ‘digital health’ OR mhealth OR telemedicine OR tele-medicine OR telephone* OR dataphone* OR videoconferenc* OR ‘video conferenc*’ OR webcast OR computer* OR questionnaire* OR survey* OR ‘compact disk’ OR ‘CD-I’ OR ‘CD-ROM’ OR DVD OR ((mobile OR portable OR educational) NEXT/2 (app OR apps OR application*)) OR website* OR ‘web site*’ OR multimedia OR ((psychosocial OR social) NEXT (care OR support OR therapy OR intervention*)) OR telegenetic OR ‘educational material*’ OR ‘tailored message*’ OR ‘message tailoring’):ab,ti) AND ((((family OR genetic* OR genomic* OR cascade OR mutation*) NEAR/3 (counsel* OR care OR testing OR screening OR analys* OR study OR studies OR discrimination OR diagnos*)) OR ‘DNA sequencing’ OR ‘preventive genetics’ OR ((family OR relative*) NEAR/3 (communicat* OR intervention* OR inform*)) OR (informed NEXT (decision* OR choice*))):ab,ti)

PsycInfo

(20200615; 123 hits)

((HBOC syndrome OR ((hereditary nonpolyposis OR hereditary non polyposis) ADJ3 (cancer OR neoplasm*)) OR HNPCC OR Lynch syndrome OR Lynch II syndrome OR Muir Torre syndrome OR BRCA* OR FANCD* OR (Fanconi anaemia ADJ3 D1) OR (MutL ADJ2 homolog 1) OR MLH1 OR hMLH1 OR MutS homolog 2 OR MSH2 OR hMSH2 OR MSH6 OR postmeiotic segregation increased protein 2 OR PMS2 OR epithelial cell adhesion molecule OR EPCAM).ab,ti. OR (oncogene OR ((cancer* OR tumor* OR tumour*) ADJ3 (syndrome OR risk OR predisposition OR susceptibility OR anticipation OR prognosis OR disorder OR gene*))).ab,ti. OR (((Genetics/AND Predisposition/) OR mutations/OR Genetic Disorders/OR At Risk Populations/OR (hereditary OR inherit* OR inborn OR familial OR mutation* OR genetic* OR ((family OR genomic*) ADJ3 (syndrome OR risk OR predisposition OR disposition OR susceptibility OR anticipation OR prognosis OR disorder OR condition* OR history)) OR single nucleotide polymorphism* OR SNP OR SNPs).ab,ti.) AND (exp neoplasms/OR (cancer* OR neoplas* OR tumor* OR tumour* OR carcinoma* OR carcinogenesis OR malignan*).ab,ti.)))

and

(counseling/OR genetic counseling/OR Professional Consultation/OR decision support systems/OR exp interpersonal communication/OR persuasive communication/OR health education/OR Interviews/OR Questionnaires/OR internet/OR exp computers/OR mobile applications/OR multimedia/OR exp telemedicine/OR social support/OR (((family OR genetic OR genomic OR patient* OR intervention OR risk*) ADJ3 counsel*) OR preventive genetics OR consultation* OR teleconsultation* OR (decision ADJ2 (support* OR aid* OR framework OR computer-assisted)) OR communicat* OR disclos* OR persuasion OR ((medical OR health OR patient* OR cancer OR risk* OR program*) ADJ3 (information OR education)) OR internet OR world wide web OR web-based OR online OR social media OR facebook OR twitter OR ((cell OR cellular OR mobile OR smart) ADJ phone*) OR cellphone* OR smartphone* OR mail OR (postal ADJ (delivery OR service)) OR letter* OR telehealth OR tele-health OR ehealth OR e-health OR digital health OR mhealth OR telemedicine OR tele-medicine OR telephone* OR dataphone* OR videoconferenc* OR video conferenc* OR webcast OR computer* OR questionnaire* OR survey* OR compact disk OR CD-I OR CD-ROM OR DVD OR ((mobile OR portable OR educational) ADJ2 (app OR apps OR application*)) OR website* OR web site* OR multimedia OR ((psychosocial OR social) ADJ (care OR support OR therapy OR intervention*)) OR telegenetic OR educational material* OR tailored message* OR message tailoring).ab,ti.)

and

(genetic counseling/OR genetic testing/OR exp family therapy/OR (((family OR genetic* OR genomic* OR cascade OR mutation*) ADJ3 (counsel* OR care OR testing OR screening OR analys* OR study OR studies OR discrimination OR diagnos*)) OR DNA sequencing OR preventive genetics OR ((family OR relative*) ADJ3 (communicat* OR intervention* OR inform*)) OR (informed ADJ (decision* OR choice*))).ab,ti.)

and

((Randomized Controlled Trial OR Controlled Clinical Trial OR Pragmatic Clinical Trial OR Equivalence Trial OR Clinical Trial, Phase III).pt. OR Randomized Controlled Trial/OR exp Randomized Controlled Trials as Topic/OR “Randomized Controlled Trial (topic)”/OR Controlled Clinical Trial/OR exp Controlled Clinical Trials as Topic/OR “Controlled Clinical Trial (topic)”/OR Randomization/OR Random Allocation/OR Double-Blind Method/OR Double Blind Procedure/OR Double-Blind Studies/OR Single-Blind Method/OR Single Blind Procedure/OR Single-Blind Studies/OR Placebos/OR Placebo/OR Control Groups/OR Control Group/OR (random* OR sham OR placebo*).ti,ab,hw. OR ((singl* OR doubl*) ADJ (blind* OR dumm* OR mask*)).ti,ab,hw. OR ((tripl* OR trebl*) ADJ (blind* OR dumm* OR mask*)).ti,ab,hw. OR (control* ADJ3 (study OR studies OR trial* OR group*)).ti,ab. OR (Nonrandom* OR non random* OR non-random* OR quasi-random* OR quasirandom*).ti,ab,hw. OR allocated.ti,ab,hw. OR ((open label OR open-label) ADJ5 (study OR studies OR trial*)).ti,ab,hw. OR ((equivalence OR superiORity OR non-inferiORity OR noninferiORity) ADJ3 (study OR studies OR trial*)).ti,ab,hw. OR (pragmatic study OR pragmatic studies).ti,ab,hw. OR ((pragmatic OR practical) ADJ3 trial*).ti,ab,hw. OR ((quasiexperimental OR quasi-experimental) ADJ3 (study OR studies OR trial*)).ti,ab,hw. OR (phase ADJ3 (III OR “3”) ADJ3 (study OR studies OR trial*)).ti,hw.)
