# Supplementary material for: Interventions Facilitating Family Communication of Genetic Testing Results and Cascade Screening in Hereditary Breast/Ovarian Cancer or Lynch Syndrome: A Systematic Review and Meta-Analysis
Source: Cancers (Basel). 2021 Feb 23;13(4):925. doi: 10.3390/cancers13040925 (PMC7926393; doi:10.3390/cancers13040925)
Supplement: Supplementary file 1 [file cancers-13-00925-s001.pdf]

Supplementary Materials: Interventions Facilitating Family Communication of Genetic Testing Results and Cascade Screening in Hereditary Breast/Ovarian Cancer or Lynch Syndrome: A Systematic Review and Meta-Analysis

Table S1. Tabular representation of risk of bias in individual studies.

Study (Author/Year)	Sequence Generation	Allocation Concealment	Blinding of Participants and Personnel	Blinding of Outcomes Accessors	Incomplete Outcome	Selective Outcome Reporting	Other Sources of Bias
Bodurtha 2014 [46]	+	+	-	?	?	?	-
Dekker 2015 [50]	+	+	-	-	+	+	?
Eijzenga 2018 [47]	+	+	+	+	+	-	-
Hodgson 2016 [48]	+	+	-	-	+	+	-
Katapodi 2018 [42]	+	?	-	?	+	+	?
Loader 2002 [51]	?	?	-	?	+	-	+
Lobb 2002 [43]	+	+	-	?	?	?	-
Mc-Inerney-Leo 2004 [41]	?	+	-	-	?	+	?
Montgomery 2013 [44]	+	?	+	?	+	+	+
Niu 2019 [52]	?	?	-	?	?	+	?
Roshanai 2009 [49]	+	?	+	+	-	?	-
Tiller 2006 [53]	+	+	+	?	+	+	?
Vogel 2019 [54]	+	+	-	-	+	+	?
Wakefield 2008 [45]	?	+	?	?	?	+	+
+ Low risk of bias	-	High risk of bias		?	Unclear risk of bias		

Copyright: © 2021 by the authors. Licensee MDPI, Basel, Switzerland. This article is an open access article distributed under the terms and conditions of the Creative Commons Attribution (CC BY) license (http://creativecommons.org/licenses/by/4.0/).
